# Probing biophysical sequence constraints within the transmembrane domains of rhodopsin by deep mutational scanning

**DOI:** 10.1126/sciadv.aay7505

**Published:** 2020-03-04

**Authors:** Wesley D. Penn, Andrew G. McKee, Charles P. Kuntz, Hope Woods, Veronica Nash, Timothy C. Gruenhagen, Francis J. Roushar, Mahesh Chandak, Chris Hemmerich, Douglas B. Rusch, Jens Meiler, Jonathan P. Schlebach

**Affiliations:** 1 Department of Chemistry, Indiana University, Bloomington, IN 47405, USA.; 2 Department of Chemistry, Vanderbilt University, Nashville, TN 37235, USA.; 3 Chemical and Physical Biology Program, Vanderbilt University, Nashville, TN 37235, USA.; 4 Center for Genomics and Bioinformatics, Indiana University, Bloomington, IN 47405, USA.

## Abstract

Membrane proteins must balance the sequence constraints associated with folding and function against the hydrophobicity required for solvation within the bilayer. We recently found the expression and maturation of rhodopsin are limited by the hydrophobicity of its seventh transmembrane domain (TM7), which contains polar residues that are essential for function. On the basis of these observations, we hypothesized that rhodopsin’s expression should be less tolerant of mutations in TM7 relative to those within hydrophobic TM domains. To test this hypothesis, we used deep mutational scanning to compare the effects of 808 missense mutations on the plasma membrane expression of rhodopsin in HEK293T cells. Our results confirm that a higher proportion of mutations within TM7 (37%) decrease rhodopsin’s plasma membrane expression relative to those within a hydrophobic TM domain (TM2, 25%). These results in conjunction with an evolutionary analysis suggest solvation energetics likely restricts the evolutionary sequence space of polar TM domains.

## Teaser

Sequence constraints associated with topological energetics restrict the evolution and mutational tolerance of membrane proteins.

## INTRODUCTION

Proteins exhibit considerable variation with respect to their tolerance of amino acid substitutions. This (in)tolerance constrains evolutionary trajectories and defines the extent to which proteins are sensitive to the effects of random genetic variation (*1*). The functional impact of some mutations can be inferred from protein structure or from evolutionary conservation. However, the fitness effects of many sequence variants arise from their impact on protein folding and solubility (*2*). The effects of mutations on the energetics of protein folding also appear to be largely responsible for the nonadditivity of most mutational fitness effects (epistasis) (*3*–*5*). Beyond the evolutionary impact of sequence variants, it should also be noted that the disruption of protein folding is the most common loss-of-function (LOF) mechanism associated with pathogenic mutations within disease-linked genes (*6*–*8*). For these reasons, efforts to rationalize and/or predict the effects of mutations on protein folding and function are of central importance to a variety of imminent challenges in both evolutionary biology (*9*) and precision medicine (*8*, *10*–*12*).

Despite decades of protein folding research, it remains exceedingly difficult to predict the effects of mutations on protein folding and stability (*13*). This is especially true for mutations within integral membrane proteins (*14*), which rely on a complex network of cellular machinery to achieve their native conformation within the membrane. Membrane protein folding is generally considered to proceed through two stages (*15*). In the first stage, the polypeptide establishes its orientation, with respect to the membrane (topology). In mammalian cells, this reaction is typically mediated by the Sec61 translocon complex during translation at the endoplasmic reticulum (ER) membrane (*10*, *16*). In the second stage, interactions between transmembrane (TM) domains drive the formation of the native conformation. The mutagenic disruption of either stage can result in the misfolding, ER retention, and premature degradation of nascent membrane proteins in the cell (*17*–*19*). However, it is interesting to consider that the energetics of these two reactions are not equally robust. Native protein structures are generally favored by 5 to 10 kcal/mol relative to unfolded ensembles, which is typically sufficient to withstand the effects of most disruptive mutations (*3*, *4*). In contrast, the translocon-mediated membrane integration of a typical TM domain within a polytopic membrane protein is close to energetically neutral, and ~25% of such TM domains are predicted to be too polar to spontaneously partition into the membrane (*20*). These considerations suggest cotranslational folding transitions should be quite sensitive to the effects of mutations, especially to those within polar TM domains. Nevertheless, it remains challenging to distinguish the effects of mutations on co- and posttranslational folding, much less how this distinction relates to evolutionary constraints within TM domains.

To explore the relationship between membrane protein folding and biosynthesis, we recently evaluated the influence of certain classes of mutations on the plasma membrane expression of rhodopsin (*18*), which is linked to the molecular basis of retinitis pigmentosa (RP) (*21*). Many rhodopsin mutations associated with RP destabilize its native structure in a manner that promotes its premature degradation and a net reduction in mature protein (*21*, *22*). Nevertheless, the structural mechanisms associated with rhodopsin misfolding remain poorly understood. Rhodopsin’s seventh TM domain (TM7) contains several functional polar residues that compromise the efficiency of its translocon-mediated membrane integration (*18*). As a result, the expression and maturation of rhodopsin are tightly linked to the hydrophobicity of this domain. Furthermore, mutations that compromise the topological energetics of TM7 exhibit a diminished proteostatic response to rhodopsin’s retinal cofactor. On the basis of these observations, we hypothesized that the kinetic and thermodynamic constraints associated with the cotranslational folding of TM7 should restrict its mutational tolerance. In this work, we use deep mutational scanning to compare the proteostatic effects of hundreds of mutations within TM7 to those within a considerably more hydrophobic TM domain (TM2). TM2 was chosen as a reference helix due to the fact that it should not perturb targeting to the ER membrane, oligomerization, or the retinal binding pocket within rhodopsin. Our results confirm that a higher proportion of missense variants within TM7 reduce the plasma membrane expression of rhodopsin. We also find that missense variants within TMs 2 and 7 exhibit notable differences in their proteostatic response to retinal. Together, our findings suggest that co- and posttranslational folding energetics may differentially constrain the evolution of TM domains.

## RESULTS

### Production of stable HEK293T cell lines expressing rhodopsin variant libraries

**Fig. 1:**
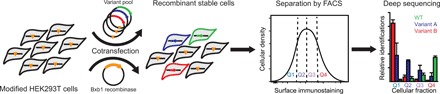
A mutational scan for the surface expression of rhodopsin variants. A cartoon depicts the general workflow for the deep mutational scanning assay described herein. A pool of stable cells expressing single rhodopsin variants from a common genomic locus is first produced by cotransfecting a plasmid library and an expression vector for Bxb1 recombinase (*23*). Recombined cells are then isolated on the basis of their characteristic bicistronic enhanced green fluorescent protein (eGFP) expression. The stable library is then fractionated according to the surface immunostaining levels of expressed rhodopsin variants using fluorescence-activated cell sorting (FACS). The relative abundance of each variant within each fraction is then evaluated by deep sequencing. Sequencing data are then used to determine the relative surface immunostaining of each variant. HEK293T, human embryonic kidney 293T.

**Fig. 2:**
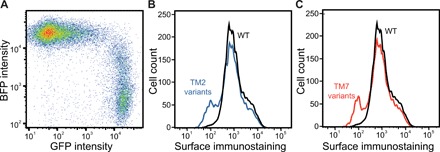
Characterization of stable cellular libraries expressing individual rhodopsin variants. Recombinant stable cell lines expressing single rhodopsin variants were characterized by flow cytometry. (**A**) A stable HEK293T cell line bearing a single genomic attP recombination site was cotransfected with a Bxb1 expression vector and a plasmid cassette bearing an attB recombination site and a library of rhodopsin variants bearing missense and nonsense mutations in TM2, and the fluorescence profiles were analyzed by flow cytometry 4 days after transfection. A dot plot shows the distribution of single-cell fluorescence intensities among transfected cells. BFP is expressed from the unmodified genomic landing pad and serves as a marker for cells that have failed to undergo recombination. GFP is expressed as a consequence of recombination between the vector and landing pad and serves as a marker for recombinant stable cells. (**B**) An intact recombinant cell line expressing missense variants within TM2 were immunostained for surface rhodopsin before analysis of cellular fluorescence profiles by flow cytometry. A histogram depicts the distribution of fluorescence intensities associated with the rhodopsin immunostaining of stable cells expressing individual TM2 variants (blue). A histogram depicting the distribution of cellular fluorescence intensities associated with surface rhodopsin levels among cells expressing wild-type (WT) rhodopsin (black) is shown for reference. (**C**) An intact recombinant cell line expressing missense variants within TM7 was immunostained for surface rhodopsin before analysis of cellular fluorescence profiles by flow cytometry. A histogram depicts the distribution of fluorescence intensities associated with the rhodopsin immunostaining of stable cells expressing individual TM7 variants (red). A histogram depicting the distribution of cellular fluorescence intensities associated with surface rhodopsin levels among cells expressing WT rhodopsin (black) is shown for reference.

To survey biosynthetic sequence constraints by deep mutational scanning, we generated cellular libraries expressing individual rhodopsin variants, fractionated the cells according to rhodopsin surface expression levels, and quantified the variants within each fraction by deep sequencing (Fig. 1). For these studies, we generated a series of stable human embryonic kidney (HEK) 293T cell lines expressing single rhodopsin variants using a recently described recombination system (*23*). Briefly, stable HEK293T cells bearing a unique genomic recombination site were cotransfected with a Bxb1 recombinase expression vector and a cassette carrying a mixed library of rhodopsin variants downstream from the cognate recombination site. Irreversible recombination between the recombination sites in the genome and cassette installs a single rhodopsin variant downstream from a genomic Tet-inducible promoter (*23*). Recombined cells, which are marked by a loss of blue fluorescent protein (BFP) expression and a gain in bicistronic enhanced green fluorescent protein (eGFP) expression (see Materials and Methods), inducibly express a single rhodopsin variant from a common genomic locus. On the basis of the percentage of eGFP+/BFP− cells, we typically achieve 20 to 40% recombination efficiency (Fig. 2A). Recombination reactions carried out in a 100-mm tissue culture dish should therefore generate several million unique clones, which is sufficient to exhaustively sample several thousand variants (fig. S1). Two recombinant cell lines, including one expressing missense variants in TM2 and one expressing missense variants in TM7, were generated in this manner and isolated by fluorescence-activated cell sorting (FACS). Rhodopsin surface immunostaining intensities associated with these two cellular libraries deviate considerably relative to those of cells expressing wild-type (WT) protein (Fig. 2, B and C). Each distribution contains a prominent shoulder at low intensities that likely represent cells expressing misfolded (class II) variants (Fig. 2, B and C). The range of mutagenic effects observed within these libraries are resolvable by flow cytometry.

### Massively parallel quantification of surface immunostaining levels of rhodopsin variants

**Fig. 3:**
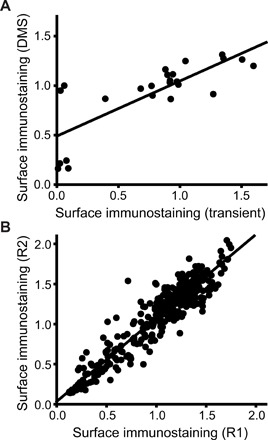
Validation and reproducibility of deep mutational scanning measurements. The accuracy and precision of surface immunostaining values determined by deep mutational scanning were assessed. (**A**) Surface immunostaining levels associated with 12 rhodopsin variants were determined in the presence and absence of 5 μM 9-cis-retinal by deep mutational scanning (*y*-coordinate), normalized by the WT value, and plotted against the corresponding values determined from a flow cytometry–based analysis of transiently expressed rhodopsin variants under the same conditions (*x*-coordinate). A linear fit of the data (Pearson’s *R* = 0.78) is included for reference. (**B**) Deep mutational scanning measurements from two independent biological replicates for TM2 variants are shown as a representative example. Surface immunostaining values for 446 rhodopsin variants bearing mutations in TM2 were measured by deep mutational scanning and normalized relative to the value for the WT protein. Values from two representative replicate experiments (R1 and R2) are plotted against one another. A linear fit of the data (Pearson’s *R* = 0.95) is shown for reference.

Recent efforts to characterize mutational tolerance within soluble proteins by deep mutational scanning have evaluated total protein expression levels by comparing the expression of GFP-tagged variants (*11*). However, a diminished plasma membrane expression is the most common effect of LOF mutations within integral membrane proteins (*10*, *19*, *24*). Therefore, to compare the proteostatic effects of these mutations, we instead chose to fractionate each cellular library into quartiles according to the intensity associated with the surface immunostaining of expressed rhodopsin variants by FACS (Fig. 1). Deep sequencing was then used to determine the relative abundance of variants within each cellular quartile. To approximate individual surface immunostaining intensities, we calculated an average intensity for each variant by weighing its number of sequence-based identifications by the fluorescence intensity of each corresponding cellular quartile (see Materials and Methods). With a few potential exceptions (fig. S2 and table S1), we observed little difference between scores for synonymous codons. We therefore grouped sequencing reads for synonymous variants according to their corresponding amino acid substitution. For ease of comparison, intensity values were normalized relative to the value of WT. Normalized intensity values averaged across two independent biological replicates are correlated with independent surface immunostaining measurements determined for a series of transiently expressed rhodopsin variants (Pearson’s *R* = 0.78; Fig. 3A), which demonstrates that these measurements are reasonably accurate. Moreover, normalized intensity values derived in this manner are generally reproducible as judged by the correlation coefficients between measurements derived from independent biological replicates for each variant library (Pearson’s *R* = 0.89 to 0.96; Fig. 3B). Together, these results validate the use of this assay for the measurement of the effects of mutations on the surface expression of rhodopsin.

### Effects of mutations on the surface expression of the opsin apoprotein

**Fig. 4:**
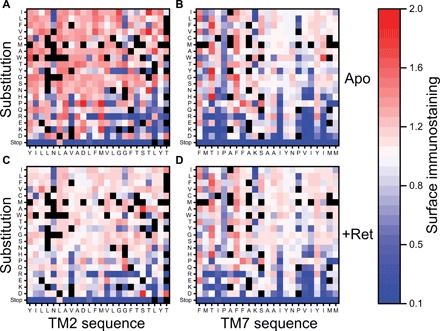
Influence of mutations within TM domains 2 and 7 on the surface immunostaining of opsin and rhodopsin. Surface immunostaning levels for rhodopsin variants bearing mutations within TMs 2 and 7 were determined by deep mutational scanning in the presence and absence of 9-cis-retinal and then normalized relative to the value of WT. Heatmaps depict the relative surface immunostaining values for opsin variants bearing each amino acid substitution (*y*-coordinate) at each position (*x*-coordinate) within TM2 (**A**) and TM7 (**B**) in the absence of retinal. Heatmaps depicting the relative surface immunostaining values for rhodopsin variants bearing each amino acid substitution within TM2 (**C**) and TM7 (**D**) in the presence of 5 μM 9-cis-retinal are also shown. Amino acids are arranged on the *y*-coordinate from the most hydrophobic (top) to the most polar (bottom) according to the White and von Heijne biological hydrophobicity scale (*39*). A value of 1.0 (white) corresponds to the surface immunostaining value for WT opsin/rhodopsin under each conditions. Variants that lack sufficient data for accurate quantification are indicated in black. Values reflect the averages from two biological replicates.

We first used this assay to evaluate the effects of mutations in TMs 2 and 7 on the surface expression of the apo-form of the protein (opsin). The heat maps in Fig. 4 display the estimated surface immunostaining intensities for each missense variant within TMs 2 and 7 normalized relative to that of WT. Many of the observed mutagenic effects are consistent with general expectations. For instance, truncations resulting from 42 of 45 nonsense mutations reduce surface protein levels by at least 75% (Fig. 4, A and B). In most cases (28 of 38 variants), mutation of the conserved prolines within TM7 (residues 291 and 303) reduces surface expression levels by at least 20% (Fig. 4B). In addition, 50% of the variants that introduce a charged residue into either TM domain reduce surface expression levels by at least 20%. Lastly, 9 of the 15 mutations within these regions that are known to cause RP exhibit diminished surface expression levels (table S2), which reflects the prominent role of rhodopsin misfolding in the molecular basis of this disease (*21*). Each of the three variants that were previously classified as misfolded (class II) variants exhibits a reduced expression level (table S2). Together, these observations demonstrate the utility of this assay for the evaluation of the proteostatic effects of mutations in rhodopsin.

Because of the intrinsic propensity of TM7 to adopt aberrant topologies (*18*), we have hypothesized that rhodopsin biosynthesis should be more sensitive to mutations in TM7 than to mutations within TM2. Perhaps unsurprisingly, we find numerous mutations within each of these domains that reduce the yield of surface opsin (Fig. 4). Nevertheless, we find that a higher proportion of mutations within TM7 (151 of 407 variants, 37%) decrease the surface expression of opsin by at least 10% relative to those within TM2 (99 of 401 variants, 25%), which is consistent with our hypothesis. It seems likely that most of these proteostatic effects stem from mutagenic perturbations of the conformational equilibrium. However, the disruptive effects of these mutations could occur at the level of cotranslational folding (topogenesis), posttranslational folding (secondary, tertiary, or quaternary structure formation), or some combination of the two. Differentiation of the types of misfolding transitions promoted by these mutations is needed to gain insights into the nature of the sequence constraints within these domains.

### Effects of mutations on the energetics of co- and posttranslational misfolding

**Fig. 5:**
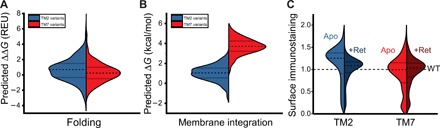
Distribution of mutagenic effects on the surface immunostaining and conformational stability of opsin and rhodopsin. Trends associated with the surface immunostaining of rhodopsin variants and their predicted energetic effects on cotranslational and posttranslational folding are shown. (**A**) Violin plots depict the statistical distribution of the effects of all possible missense mutations within TM2 (480 total, blue) and TM7 (460 total, red) on the energetics of rhodopsin folding in Rosetta energy units as calculated using the RosettaMembrane energy scoring function (*25*). The shape of each distribution was defined using a kernel smoothing function. Dashed lines within the violins reflect the median value, while dotted lines within the violins reflect the positions of the 25th and 75th percentiles. Positive ΔΔ*G* values are indicative of a destabilization of the native structure. (**B**) Violin plots depict the statistical distribution of the effects of all possible missense mutations within TM2 (480 total, blue) and TM7 (460 total, red) on the translocon-mediated membrane integration of the corresponding transmembrane domains, which were calculated using the Δ*G* predictor (*20*). The shape of each distribution was defined using a kernel smoothing function. Dashed lines within the violins reflect the median value, while dotted lines within the violins reflect the positions of the 25th and 75th percentiles. Positive Δ*G* values indicate the translocon-mediated membrane integration of the helix is unfavorable. (**C**) Violin plots depict the statistical distribution of surface immunostaining values associated with missense mutations within TM2 (401 total, left) or TM7 (407 total, right) in the presence (dark blue, dark red) and absence (light blue, light red) of 5 μM 9-cis-retinal. Immunostaining intensity values were determined by deep mutational scanning and were normalized relative to the value for the WT protein. The shape of each distribution was defined using a kernel smoothing function. Dashed lines within the violins reflect the median value, while dotted lines within the violins reflect the positions of the 25th and 75th percentiles. Values reflect the averages from two biological replicates.

The divergent proteostatic effects of mutations within these TM domains could potentially arise from intrinsic differences in the energetic contributions of each TM domain to the stability of the native conformation. If the disparate proteostatic effects within these TM domains primarily arise from disruption of the native structure, then we should expect a higher proportion of mutations within TM7 to increase the free energy of folding. Unlike current experimental approaches, computational measurements allow us to explicitly dissect the energetic effects of mutations on the topological energetics (stage I folding) from their effects on the stability of the native rhodopsin fold (stage II folding). Therefore, to evaluate whether mutations in either TM domain are more likely to destabilize the native fold, we used the Rosetta ΔΔ*G* protocol to estimate the energetic effects of all possible missense mutations on the stability of the native conformation (ΔΔ*G*) based on the structure of rhodopsin (*25*). Although there are quantitative limitations associated with stability predictions for individual variants (*14*), ensembles of stability predictions like these have been previously used to identify general trends associated with mutagenic perturbations of protein structure (*9*, *26*). The distribution of Rosetta ΔΔ*G* values associated with mutations within these domains is quite similar (Fig. 5A). Slight differences in the median ΔΔ*G* values suggest that, if anything, a lower proportion of mutations within TM7 should be destabilizing relative to those within TM2 (Fig. 5A). These data suggest that the increased proportion of disruptive mutations within TM7 is unlikely to reflect an underlying disparity in the energetic effects of mutations on the posttranslational folding of rhodopsin.

Although mutagenic effects within TMs 2 and 7 appear to be more or less symmetric, with respect to their effects on the stability of the native conformation, these mutations may differ with respect to their effects on the fidelity of cotranslational folding. If the disparate proteostatic effects within these TM domains arise from disruption of cotranslational membrane integration, then we should expect a higher fraction of mutations within TM7 to increase the free energy associated with its transfer from the translocon to the membrane. To evaluate the extent to which mutations disrupt the topological energetics within each domain, we used a knowledge-based algorithm (Δ*G* predictor) (*20*) to estimate the transfer free energy associated with the cotranslational membrane integration of each helical variant. The distribution of predicted transfer free energies associated with missense variants of each TM domain is similar in breadth (Fig. 5B), which suggests that the energetic effects of mutations on the transfer free energy of each helix (ΔΔ*G* values) are similar in magnitude. However, because of the underlying difference in the hydrophobicity of these domains, the median transfer free energy among TM7 variants (+3.7 kcal/mol) is higher than that of the TM2 variants (+1.1 kcal/mol) (Fig. 5B). Given that cotranslational membrane integration of WT TM7 is known to be inefficient (*18*), mutagenic effects within TM7 are perhaps more likely to change the fraction of native topomer relative to those within TM2. This energetic discrepancy in cotranslational folding energetics could therefore contribute to asymmetric proteostatic effects of mutations within these two TM domains.

Our previous findings on the cotranslational misfolding of TM7 provide an additional lens for the interpretation of mutagenic effects within TM7. We recently demonstrated that mutations within TM7 promote the formation of two competing non-native topomers during cotranslational folding, including one in which K296 snorkels into the membrane interface (near-native topomer) and one in which TM7 slips into the ER lumen (misfolded topomer; fig. S3A) (*18*). Mutations that promote the formation of these aberrant topomers have been found to decrease the surface expression of rhodopsin (*18*). A comparison of these putative topological states reveals that the eight C-terminal residues of TM7 (residues 302 to 309) are embedded within the membrane in the native topomer but solvated by water in these two non-native topological states (fig. S3A). On the basis of the differential solvation of these residues (fig. S3A), we expect that the introduction of charged residues at these positions should promote the formation of aberrant topomers in a manner that enhances opsin misfolding. Variants that introduce a non-native charge at these positions (residues 302 to 309) exhibit a lower median surface expression level (median = 0.56, σ = 0.40, *n* = 33), relative to those bearing charged residues in the N-terminal portion of TM7 (residues 287 to 301, median = 0.78, σ = 0.44, *n* = 55; fig. S3B). This observation provides additional evidence that the proteostatic effects of certain mutations within TM7 are likely to arise from their disruption of cotranslational folding transitions.

### Asymmetric influence of retinal on the surface expression of rhodopsin variants

Rhodopsin binds its retinal cofactor with an estimated *K*_d_ (dissociation constant) of 25 pM (*27*), and the stabilization afforded by the thermodynamic coupling between binding and folding is known to enhance the cellular expression of rhodopsin (*28*). However, topological defects in TM7 prevent the formation of the native retinal binding pocket (*18*). As a result, the stabilization afforded by retinal binding cannot compensate for the proteostatic effects of mutations that disrupt the topology of TM7 (*18*). If the heightened sensitivity of TM7 to the effects of mutations arises from their influence on the fidelity of cotranslational folding, then we would expect fewer of misfolded TM7 variants to respond to retinal. To compare the proteostatic effects of mutations within TMs 2 and 7 in the presence of cofactor, we repeated our deep mutational scan in the presence of 5 μM 9-cis-retinal, which is a photostable isomer of rhodopsin’s native chromophore. Many of the mutagenic effects observed for the opsin apoprotein persist in the presence of retinal (Fig. 4). For instance, almost every nonsense mutation reduces expression levels under these conditions (Fig. 4, C and D). However, in contrast to the mutational scan of the apoprotein, the relative surface immunostaining levels of most TM2 variants appear indistinguishable from WT in the presence of retinal (Figs. 4, A and C, and 5C). This compression of variant scores arises from the fact that the WT surface immunostaining also increases in response to retinal (*18*). Mutations that increase the stability of the apoprotein exhibit a smaller increase in expression relative to WT in the presence of retinal and vice versa. This observation implies the energetic effects of these mutations on the conformational equilibrium are small relative to that of retinal. The fact that retinal appears to compensate for mutagenic effects within TM2 suggests mutations within this domain primarily perturb reactions that are thermodynamically coupled to binding, such as those involved in posttranslational folding of the native topomer. In contrast, most of the proteostatic effects associated with TM7 variants persist in the presence of retinal (Figs. 4, B and D, and 5C), which is an emergent property of variants that induce topological defects (*18*). The inability of retinal to buffer the proteostatic effects of mutations within TM7 provides additional evidence suggesting that the propensity of these mutations to promote cotranslational misfolding contributes to the asymmetric proteostatic effects of mutations within these domains.

### Attenuated evolution of residues within TM7

**Fig. 6:**
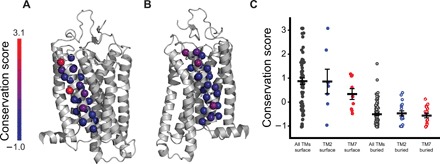
Evolutionary profiling of residues within the transmembrane domains of rhodopsin. The sequences of 468 rhodopsins were aligned and analyzed to compare the evolutionary rates of residues within each TM domain. (**A**) The evolutionary rates associated with each residue within TM2 were converted into conservation scores and mapped onto a structural model of rhodopsin. (**B**) The evolutionary rates associated with each residue within TM7 were converted into conservation scores and mapped onto a structural model of rhodopsin. (**C**) The conservation scores associated with the surface (closed circles) and buried (open circles) residues within all of the TM domains of rhodopsin (gray) are compared to those within TMs 2 (blue) and 7 (red), specifically. The average conservation score associated with each distribution is plotted, along with whiskers showing the standard error plotted for reference.

Regardless of the mechanism, the disparity associated with the proteostatic effects of mutations within TMs 2 and 7 is more pronounced in the presence of retinal, which is abundant under physiological conditions. The reduced mutational tolerance of TM7 likely limits its accessible sequence space. To assess whether the evolutionary divergence of TM7 appears to be restricted relative to hydrophobic TM domains, we used ConSurf to compare the conservation of residues within the TM domains of rhodopsin (*29*). ConSurf generates a conservation score for each position, which reflects how many SDs its evolutionary rate is from that of the mean. These scores facilitate quantitative comparisons of the relative variability (positive values, red) or conservation (negative values, blue) of each residue. Conservation scores generated from an analysis of 468 rhodopsin sequences suggest that, like those within other TM domains, buried residues within TMs 2 and 7 are highly conserved (Fig. 6). The conservation of buried residues likely reflects their contributions to the native structure and function of rhodopsin. In contrast, residues exposed to the lipid bilayer are more variable (Fig. 6). While the range of conservation scores for surface residues within TM2 is comparable to those within other TM domains, scores for the exposed residues within TM7 appear to be somewhat lower (Fig. 6C). These evolutionary patterns must be influenced by a number of constraints associated with both rhodopsin folding and function. Nevertheless, the attenuated variability observed within the surface residues of TM7 may, in part, reflect the reduced number of permissible substitutions within this domain.

## DISCUSSION

The impacts of mutations on the fidelity of protein folding make substantial contributions to protein fitness and evolution (*3*, *4*, *9*). The kinetic and thermodynamic barriers that govern the folding of integral membrane proteins are distinct from those of soluble proteins (*10*). Moreover, membrane proteins rely on a distinct portion of the proteostasis network that imposes a unique set of sequence constraints (*10*). These mechanistic distinctions may therefore differentially constrain the evolutionary trajectories of integral membrane proteins. To survey the constraints of rhodopsin biosynthesis, we used deep mutational scanning to compare the proteostatic effects of missense variants within a semipolar TM domain (TM7) to those within a more hydrophobic TM domain (TM2). Our results reveal that TM7 has an attenuated mutational tolerance relative to TM2. The proteostatic effects associated with individual mutations within either domain may arise from their influence on a spectrum of conformational transitions. Nevertheless, a variety of observations suggest the limited mutational tolerance of TM7 may largely arise from the propensity of this domain to undergo cotranslational misfolding (*18*). These findings suggest that mutagenic disruptions within these domains are likely to promote the formation of distinct misfolded states that vary with respect to their response to retinal. The resulting inability of retinal to compensate for the destabilizing effects of certain mutations must affect evolutionary sequence space. An analysis of natural rhodopsin sequences reveals that the solvent-exposed residues within TM7 exhibit less variation relative to those within other TM domains, which potentially arises as a result of the hydrophobicity constraints associated with its translocon-mediated membrane integration. Together, these observations provide insights into the manner by which the energetics of membrane protein folding may shape their evolutionary trajectories.

In conjunction with other recent investigations (*11*, *30*–*32*), our findings highlight deep mutational scanning as a powerful approach to probe the interface between membrane protein folding, cellular quality control, and mutational tolerance. Future investigations are needed to determine how these biosynthetic constraints are balanced within other TM helices and how they relate to the constraints of membrane protein function. Last, it should be noted that this mutational scan appears to be capable of detecting the effects of misfolded RP variants and measuring their response to retinal (table S2). This observation highlights the potential utility of deep mutational scanning in the development and targeting of precision therapeutics (*10*).

## MATERIALS AND METHODS

### Plasmid preparation and mutagenesis

A previously described pcDNA5 vector containing the human rhodopsin complementary DNA (cDNA) bearing an N-terminal hemagglutinin (HA) tag (extracellular) followed by an internal ribosome entry site and a dasher GFP (*18*) was further modified for compatibility with a previously described recombination system (*23*). For the properties of expressed rhodopsin variants to be related back to their corresponding genetic sequence, rhodopsin must only be expressed upon recombination into the genomic “landing pad” of the stable HEK293T cell line used herein (see below) (*23*). To facilitate this conditional expression of rhodopsin, we replaced the cytomegalovirus promoter upstream of the rhodopsin cDNA with an attB recombination site using Gibson assembly. Bxb1-mediated recombination between this attB site in the plasmid and the genomic attP site in these cells will therefore install a single rhodopsin cDNA downstream from a Tet-inducible promoter (*23*). Pools of rhodopsin variants bearing every possible amino acid substitution within TMs 2 and 7 were then generated in the background of this expression vector. Briefly, a series of 47 individual site-directed mutagenesis reactions were performed using forward and reverse primers bearing randomized (NNN) bases at each codon within these TM domains. Each individual mutagenesis reaction was then transformed into chemically competent XL1-blue cells (Agilent Technologies, Santa Clara, CA), which were then grown in liquid culture overnight. Mutagenized plasmid pools (64 possible variants per reaction) were then extracted from each liquid culture. Equivalent amounts of each miniprep plasmid pool were then combined to create two libraries—one containing all codon swaps within TM2 (1512 genetic variants, 480 substitutions) and one containing codon swaps within TM7 (1449 genetic variants, 460 substitutions). Pooled libraries were then electroporated into electrocompetent NEB10β cells (New England Biolabs, Ipswitch, MA), grown in liquid culture overnight, and purified using a ZymoPure endotoxin-free midiprep kit (Zymo Research, Irvine, CA). Plasmid libraries were found to contain 929 of 940 possible coding variants by deep sequencing. Bxb1 recombinase was expressed using the pCAG-NLS-HA Bxb1 expression vector, which was provided by D. Fowler.

### Production and fractionation of recombinant cell lines

Recombinant stable cell lines expressing single rhodopsin variants were created using a previously described stable HEK293T cell line containing a genomic Tet-Bxb1-BFP landing pad that was provided by D. Fowler (*23*). Briefly, 3.5 million cells were plated within a 100-mm tissue culture dish and grown overnight in complete Dulbecco’s modified Eagle medium (Gibco, Carlsbad, CA) supplemented with 10% fetal bovine serum (Corning, Corning, NY), penicillin (100 U/ml)/streptomycin (100 μg/ml) (complete media) at 37°C in a humidified incubator containing 5% CO_2_ by volume. Cells were cotransfected using Fugene 6 transfection reagent (Promega, Madison, WI) on the following day (50 to 70% confluence) with a plasmid mixture containing the rhodopsin library and the Bxb1 recombinase expression vector (15:1 ratio, respectively). Transfections were typically carried out by mixing 7.6 μg of total plasmid with 29 μl of Fugene 6 in accordance with the manufacturer’s instructions. Transfected cells were then grown at 33°C for 4 days in full media. Doxycycline (2 μg/ml) was added to the media 1 day after transfection to induce expression of rhodopsin and eGFP. Stable cells expressing single rhodopsin variants were then isolated using a BD FACS Aria II (BD Biosciences, Franklin Lakes, NJ) 4 days after transfection based on their characteristic gain in bicistronic eGFP expression and coincident loss of BFP expression (Fig. 2A), which occurs as a result of the recombination of the rhodopsin vector into the genomic landing pad (*23*). Recombined cells were then expanded in a 100-mm tissue culture dish in complete media containing doxycycline (2 μg/ml) for 7 days. For experiments carried out in the presence of retinal, 5 μM 9-cis-retinal was added to the media on the final day before characterization of cellular libraries. To separate cells according to the surface expression levels of expressed rhodopsin variants, stable cell libraries were then harvested and immunostained using an Alexa Fluor 647–conjugated anti-HA antibody (ThermoFisher, Waltham, MA). FACS was then used to fractionate the cellular library into quartiles according to their fluorescence intensities associated with the surface immunostaining of expressed rhodopsin variants. We typically sorted ~2 million cells into each quartile to ensure exhaustive sampling of low-abundance variants. Each fraction was then expanded in a 100-mm culture dish before harvesting and freezing cell pellets (8 to 12 million cells per quartile) for genetic analysis.

### Extraction of genomic DNA and preparation of next-generation sequencing libraries

To facilitate the identification of recombined rhodopsin variants within each cellular fraction, we first extracted the genomic DNA (gDNA) from cell pellets using either a previously described protocol (*33*) or a GenElute Mammalian Genomic DNA Miniprep kit (Sigma-Aldrich, St. Louis, MO). We then used a previously described nested polymerase chain reaction (PCR) approach to selectively amplify recombined rhodopsin variants from within the gDNA (*23*). Briefly, the recombined rhodopsin cassette was first selectively amplified from the gDNA using HiFi HotStart ReadyMix (Kapa Biosytems, Wilmington, MA) in combination with a primer that anneals to the genomic landing pad and a primer that anneals within the rhodopsin cassette. To minimize PCR bias, eight replicate PCRs each containing 2.5 μg of gDNA template were carried out for seven cycles as described previously (*23*). PCR products were then purified using a DNA Clean & Concentrator-5 kit (Zymo Research, Irvine, CA), and replicate reactions were pooled. This PCR product was then used as a template for an additional PCR amplification to generate amplicons containing the mutagenized portions of the rhodopsin cDNA flanked by Illumina adapter sequences. For each cellular fraction, four replicate reactions were carried out using HiFi HotStart ReadyMix (Kapa Biosytems, Wilmington, MA), 10% of the purified PCR product template, and two primers containing Illumina adapter sequences—one that anneals outside of the mutagenized region and another that anneals to the tail of an outside primer used for the first round amplification. To minimize PCR bias, reactions were monitored by real-time PCR and terminated upon reaching mid-log amplification (typically ~20 cycles). Replicate reactions were then pooled and gel purified using a Zymoclean Gel DNA Recovery Kit (Zymo Research, Irvine, CA). The purity and concentrations of the final amplicons were evaluated using an Agilent 2200 TapeStation (Agilent Technologies, Santa Clara, CA). In some cases, higher- and lower-weight DNA was subsequently removed using the Select-a-Size DNA Clean & Concentrator Magnetic Bead System (Zymo Research, Irvine CA). Amplicons were sequenced using a NextSeq 500 Mid Output 150-cycle kit to an average depth of ~2 million reads per library.

### Estimation of surface immunostaining levels from deep mutational scanning data

To estimate surface immunostaining levels for each variant from sequencing data, we created a computational pipeline for the analysis of demultiplexed sequencing data. Briefly, next-generation sequencing data from each cellular isolate were first filtered to remove any reads in which the expected number of errors is ≥1 (*34*). We also removed any reads with an average quality score less than 30 and any reads containing mutations within multiple codons. Our quality filter removed between 7 and 20% of the reads from our raw data, and 7 to 9% of the remaining reads were removed because they contained multiple mutations. The remaining sequencing reads derived from each cellular isolate were grouped according to their corresponding amino acid substitution and then rarefied to create subsampled datasets of uniform size for each sample. To infer immunostaining levels for each variant, we calculated a weighted-average fluorescence intensity value for each variant using Eq. 1

$${\langle I\rangle }_{\text{variant}}=\frac{\sum_{i=1}^{4}{\langle F\rangle }_{i}N_{i}}{\sum_{i=1}^{4}N_{i}}$$ (1)

where ⟨*I*⟩_variant_ is the weighted-average fluorescence intensity value of a given variant, ⟨*F*⟩*_i_* is the mean fluorescence intensity associated with cells sorted into the *i*th FACS quartile, and *N_i_* is the number of reads of the variant in the *i*th FACS quartile. To facilitate meaningful comparisons of variant intensity values, weighted-average intensities calculated for each variant were then normalized by the value determined for the WT protein. To remove variant scores with insufficient sampling, we took several measures to remove outliers. Correlations between intensity values derived from independent rarefactions of each dataset were fitted to an ordinary least squares model using the statsmodels Python library, and outliers were removed on the basis of a Bonferroni correction (α = 0.05). We also removed intensity values derived from 50 or fewer reads across the four quartiles, as well as scores for any variants that were not consistently observed within each replicate of each experiment. The average number of reads associated with each variant across two biological replicates is shown in tables S3 and S4 for reference.

### Computational predictions of the effects of mutations on folding energetics

The effects of each mutation on the thermodynamic stability of rhodopsin were estimated computationally using a membrane protein–optimized Rosetta energy function in conjunction with Rosetta ΔΔ*G* protocol as was described previously (*25*). Briefly, a previously published homology model of inactive human rhodopsin based on a high-resolution structure of bovine rhodopsin [Protein Data Bank (PDB) 3C9L] (*18*) was used to generate an initial structural ensemble (50 iterations) and a corresponding average energy score for both the WT and for each variant. The ΔΔ*G* for each variant is then calculated as ΔΔ*G* = Δ*G*_Mut_ − Δ*G*_WT_. The influence of mutations on the cotranslational membrane integration of TMs 2 and 7 was estimated using the Δ*G* predictor, as was described previously (*20*).

### Structural and evolutionary calculations

To compare the evolutionary divergence of residues within TMs 2 and 7, the sequences of 468 nonredundant rhodopsin homologs were identified within the UniRef90 database using PSI-BLAST (Position Specific Iterative Basic Local Alignment Search Tool) with an *e*-value threshold of 1 × 10^−4^ (*35*). Redundant sequences were defined as those with ≥95% sequence identity and were removed using cd-hit (*36*). Nonredundant rhodopsin sequences were aligned using MAFFT (Multiple Alignment using Fast Fourier Transform) (*37*), and conservation scores were generated for each residue from the aligned sequences using ConSurf (*29*). Relative accessible surface areas for each residue were calculated using a previously published homology model of inactive human rhodopsin based on a high-resolution structure of bovine rhodopsin (PDB 3C9L) (*18*) using NACCESS with a default reference van der Waals radii (*38*). A cutoff of 20% relative accessible surface area was chosen as the criterion for defining a residue as buried or exposed.

## Supplementary Material

http://advances.sciencemag.org/cgi/content/full/6/10/eaay7505/DC1

Download PDF

Probing biophysical sequence constraints within the transmembrane domains of rhodopsin by deep mutational scanning

## SUPPLEMENTARY MATERIALS

Supplementary material for this article is available at http://advances.sciencemag.org/cgi/content/full/6/10/eaay7505/DC1 Fig. S1. Sampling of rhodopsin variants within recombinant cell lines. Fig. S2. Nucleotide-level analysis of deep mutational scanning data. Fig. S3. Topological context of mutagenic effects within TM7. Table S1. Potential nonequivalent codon substitutions. Table S2. Surface immunostaining of pathogenic rhodopsin variants. Table S3. Average number of TM2 variant reads across two biological replicates. Table S4. Average number of TM7 variant reads across two biological replicates.

## Appendix

View/request a protocol for this paper from *Bio-protocol*.
