# Supplementary material for: Probing biophysical sequence constraints within the transmembrane domains of rhodopsin by deep mutational scanning
Source: Sci Adv. 2020 Mar 4;6(10):eaay7505. doi: 10.1126/sciadv.aay7505 (PMC7056298; doi:10.1126/sciadv.aay7505)
Supplement: http://advances.sciencemag.org/cgi/content/full/6/10/eaay7505/DC1 [file supp_6_10_eaay7505__index.html]

Science Advances | Science AdvancesAAASSearchScience AdvancesMenu

## Supplementary Materials

**This PDF file includes:**

- Fig. S1. Sampling of rhodopsin variants within recombinant cell lines.
- Fig. S2. Nucleotide-level analysis of deep mutational scanning data.
- Fig. S3. Topological context of mutagenic effects within TM7.
- Table S1. Potential nonequivalent codon substitutions.
- Table S2. Surface immunostaining of pathogenic rhodopsin variants.
- Table S3. Average number of TM2 variant reads across two biological replicates.
- Table S4. Average number of TM7 variant reads across two biological replicates.

Download PDF

**Files in this Data Supplement:**

- Adobe PDF - aay7505\_SM.pdf
