# Supplementary material for: Probing biophysical sequence constraints within the transmembrane domains of rhodopsin by deep mutational scanning
Source: Sci Adv. 2020 Mar 4;6(10):eaay7505. doi: 10.1126/sciadv.aay7505 (PMC7056298; doi:10.1126/sciadv.aay7505)
Supplement: Download PDF [file aay7505_SM.pdf]

Science Advances
AAAS

advances.sciencemag.org/cgi/content/full/6/10/eaay7505/DC1

Supplementary Materials for

Probing biophysical sequence constraints within the transmembrane domains of rhodopsin by deep mutational scanning

Wesley D. Penn, Andrew G. McKee, Charles P. Kuntz, Hope Woods, Veronica Nash, Timothy C. Gruenhagen, Francis J. Roushar, Mahesh Chandak, Chris Hemmerich, Douglas B. Rusch, Jens Meiler, Jonathan P. Schlebach*

*Corresponding author. Email: jschleba@indiana.edu

Published 4 March 2020, *Sci. Adv.* **6**, eaay7505 (2020)
DOI: 10.1126/sciadv.aay7505

This PDF file includes:

Fig. S1. Sampling of rhodopsin variants within recombinant cell lines.
Fig. S2. Nucleotide-level analysis of deep mutational scanning data.
Fig. S3. Topological context of mutagenic effects within TM7.
Table S1. Potential nonequivalent codon substitutions.
Table S2. Surface immunostaining of pathogenic rhodopsin variants.
Table S3. Average number of TM2 variant reads across two biological replicates.
Table S4. Average number of TM7 variant reads across two biological replicates.

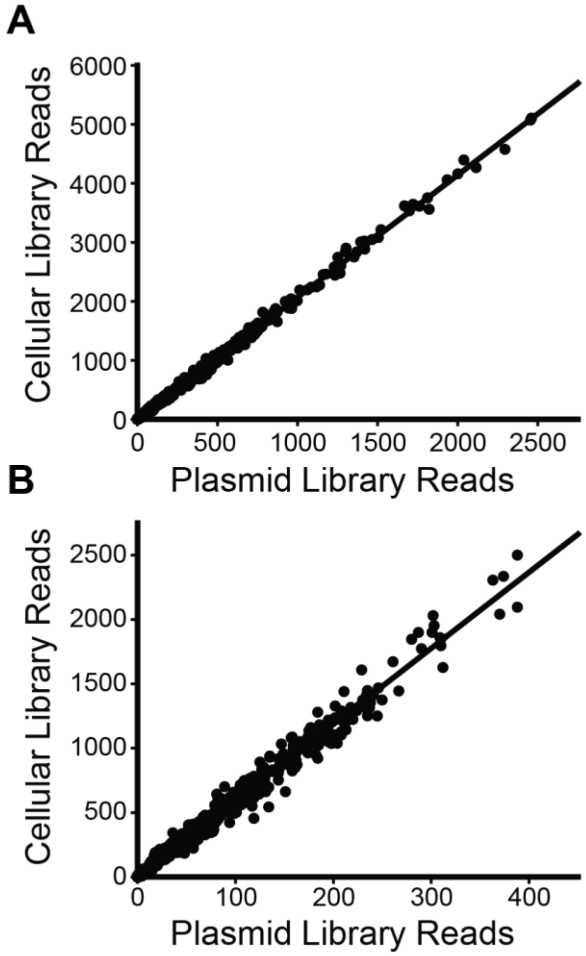

Fig. S1. Sampling of rhodopsin variants within recombinant cell lines. The relative abundance of variants within recombined cell lines was compared to that within the parental plasmid library in order to assess whether the entire spectrum of variants is sampled. A) The genomic DNA was extracted from recombinant cells expressing TM2 variants, and the mutagenized region was analyzed by next generation sequencing (NGS). The number of reads for each variant within the cellular population is plotted against the number of reads for the corresponding variant within the plasmid pool used to generate the stable cells. A linear fit of the data (Pearson's R = 0.99) is shown for reference. B) The genomic DNA was extracted from recombinant cells expressing TM7 variants, and the mutagenized region was analyzed by NGS. The number of reads for each variant within the cellular population is plotted against the number of reads for the corresponding variant within the plasmid pool used to generate the stable cells. A linear fit of the data (Pearson's R = 0.99) is shown for reference. The quantitative agreement in the relative abundance of each variant within plasmids and cellular population is taken as an indication that the library of variants is sampled completely.

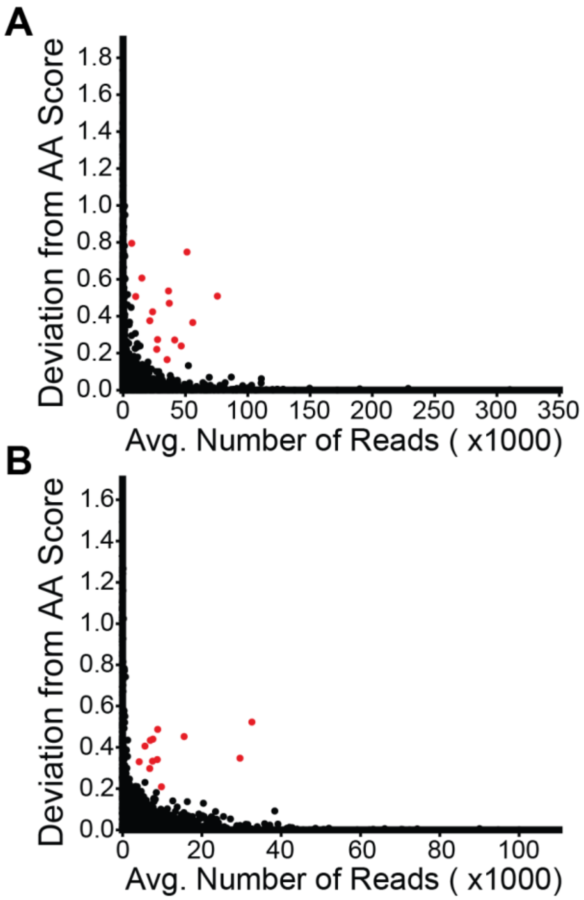

Fig. S2. Nucleotide-level analysis of deep mutational scanning data. Relative surface immunostaining values were determined for each individual codon substitution, and the difference between this value and the averaged score for equivalent coding substitutions was determined. This deviation between individual codon scores and averaged amino acid scores is plotted against the average number of NGS based identifications of the corresponding codon substitutions within the (A) TM2 and (B) TM7 libraries across two biological replicates. Well-sampled codon substitutions that exhibit little deviation from amino acid scores fall along the X-axis. Codon substitutions that exhibit variations that can potentially be attributed to poor sampling fall along the Y-axis. Codon substitutions exhibiting deviations that cannot be explained by poor sampling fall off axis (highlighted in red). A list of specific codon substitutions that could potentially have non-equivalent effects to other synonymous mutations can be found in Table S1.

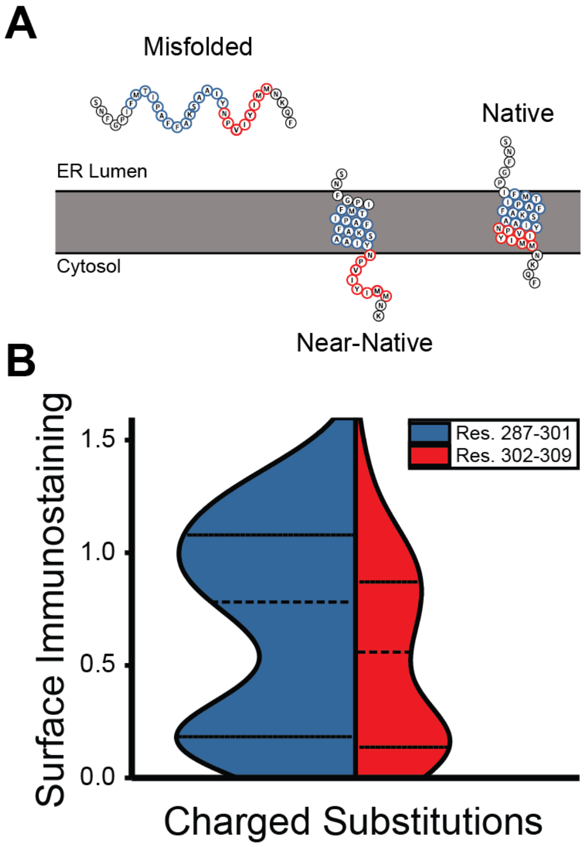

Fig. S3. Topological context of mutagenic effects within TM7. A) A cartoon depicts the three putative topological states that form during rhodopsin biosynthesis.(18) Residues 287-301 are solvated by water in the misfolded topomer and within the membrane in the native and near native topomers. Alternatively, residues 302-309 are only solvated within the membrane in the native topomer. B) A violin plot depicts the statistical distribution of the relative opsin surface immunostaining values associated with variants bearing charged substitutions within residues 287-301 relative to that of variants bearing charged substitutions within residues 302-309. The shape of each distribution was defined using a kernel smoothing function. Dashed lines within the violins reflect the median value, while solid lines within the violins reflect the positions of the 25th and 75th percentiles. Values reflect the averages from two biological replicates.

Table S1. Potential nonequivalent codon substitutions.

Mutation	Averaged AA Score*	Substitution	Codon Score**	Codon Score- AA Score
I75R	0.58	ATC→CGT	0.86	0.27
I75R	0.58	ATC→CGG	0.16	-0.42
L76R	0.79	CTG→AGG	1.30	0.51
V81E	0.34	GTG→GAA	0.56	0.22
V81E	0.34	GTG→GAG	0.17	-0.17
A82T	1.54	GCT→ACC	2.34	0.79
D83R	1.09	GAC→CGA	1.70	0.61
D83R	1.09	GAC→AGA	0.55	-0.54
D83R	1.09	GAC→AGG	1.46	0.37
T92I	0.76	ACC→ATC	1.23	0.47
T92I	0.76	ACC→ATA	0.39	-0.37
T94A	1.28	ACC→GCT	1.52	0.24
T94A	1.28	ACC→GCC	1.01	-0.27
T94S	0.91	ACC→TCT	1.41	0.51
T94S	0.91	ACC→TCA	0.16	-0.75
T289L	1.43	ACC→CTT	0.98	-0.45
T289L	1.43	ACC→CTC	1.64	0.21
I290P	0.85	ATC→CCA	1.19	0.34
F293C	1.51	TTC→TGT	1.18	-0.33
P303R	0.64	CTT→CGT	1.07	0.43
P303R	0.64	CTT→CGA	0.11	-0.52
P303R	0.64	CTT→CGG	0.97	0.33
P303R	0.64	CTT→AGG	0.98	0.35
M309I	0.76	ATG→ATT	0.28	-0.49
M309I	0.76	ATG→ATC	1.06	0.30
M309I	0.76	ATG→ATA	1.17	0.41
M309S	0.88	ATG→AGC	0.44	-0.44

*These values are average relative surface immunostaining measurements from two deep mutational scanning measurements in which all codon substitutions were grouped according to their corresponding amino acid substitution.
** These values are average relative surface immunostaining measurements from two deep mutational scanning measurements in which all codon substitutions were analyzed independently.

Table S2. Surface immunostaining of pathogenic rhodopsin variants.

Variant	TM domain	Opsin Surface Immunostaining (No Ret)	Rhodopsin Surface Immunostaining (+ 9-*cis*-Ret)	Current Classification*	Pathology
N78I	2	0.21	0.36	-	Retinitis Pigmentosa†
L79P	2	1.11	1.08	-	Retinitis Pigmentosa†
V87D	2	0.28	0.45	-	Retinitis Pigmentosa type 4
L88P	2	0.37	0.70	-	Likely Pathogenic
G89D	2	0.45	0.46	II	Retinitis Pigmentosa type 4
G90D	2	1.30	0.89	IV, VI	Congenital Night Blindness†
T92I	2	0.76	0.98	-	Retinitis Pigmentosa
T94I	2	1.78	1.14	VI	Congenital Night Blindness†
T97I	2	0.94	1.42	-	Retinitis Pigmentosa†
T289P	7	0.12	0.24	-	Retinitis Pigmentosa†
A292E	7	1.11	1.25	VI	Congenital Night Blindness†
A295V	7	1.20	1.12	VI	Congenital Night Blindness†
K296E	7	0.85	0.83	II, VI	Retinitis Pigmentosa type 4
S297R	7	0.16	0.21	II	Retinitis Pigmentosa†
A298D	7	0.78	0.96	-	Retinitis Pigmentosa

* Type II variants enhance cellular misfolding. Type IV variants exhibit modifications in post-translational modifications. Type VI variants are constitutively active.
† These variants exhibit autosomal dominant patterns of inheritance.

Table S3. Average number of TM2 variant reads across two biological replicates.

A. TM2 Variants in the Absence of Retinal

	Y74	I75	L76	L77	N78	L79	A80	V81	A82	D83	L84	F85	M86	V87	L88	G89	G90	F91	T92	S93	T94	L95	Y96	T97
**I**	45336	28461	433	11765	18523	9368	47357	18080	42732	5433	42391	24177	13385	28158	8673	43669	18422	147829	98574	1952	10292	235821	27922	29096
**L**	55410	143438	83240	256556	240323	208896	88456	6016	66180	40066	30305	46215	76739	14410	48304	207688	93595	193407	66767	37001	159623	4688	33994	66
**F**	31650	6049	28156	29107	70576	55252	6966	12	29142	17761	7007	3412	21771	777	13790	80849	44667	65988	56963	90872	851	48794	30310	2
**V**	474	11610	2544	24784	31	9395	9291	63399	30049	39449	79126	45976	22697	29373	37864	1000	143639	74388	4728	10155	22746	577	11342	45
**C**	28998	219	27669	71	67426	22210	57907	8045	66497	12631	13492	342	19549	15976	24	1047	1879	1622	103	16575	2906	24146	793	6675
**M**	10	54234	17478	17	264	1445	28	2724	14197	84	21042	418	0	23395	36027	31	6989	393	275	17	449	69169	207	4
**A**	60285	16807	2186	140417	317	35976	23549	35298	15063	32777	46580	34541	24884	45895	7251	123788	23460	299	35087	1948	88894	144117	33274	143662
**W**	11224	21	67190	0	20	64	5	11077	8342	56346	172	19365	7318	15	9106	961	65003	73157	8693	49	91032	7741	625	54
**T**	62351	67012	153413	43134	1919	83415	54584	7107	24433	15010	18459	22066	17641	26475	24226	11523	40548	135969	18961	1654	3584	272	11239	3342
**Y**	6069	115140	276	38	802	11571	7696	16	14722	19640	3158	5026	4409	637	74163	186	107535	54579	241	85	14652	56	1165	38
**G**	106946	42634	56323	40	22	6	36870	42664	49158	23462	42541	17085	56503	13759	32299	7784	63561	44073	43672	199249	25	54407	39754	18875
**S**	3876	13139	97984	149	147676	107748	60877	35428	49304	42478	60684	20532	69627	59974	69224	73023	109285	40765	144660	50422	135899	7910	29358	2725
**N**	87877	48578	125	23	803	22042	24321	1964	198	15519	15308	50111	25082	59986	270	15688	111	1131	32079	6260	28735	123	24438	2413
**H**	1648	120471	61517	20822	644	81	19456	197	42044	11905	37276	24067	8816	34501	12631	71209	34	10696	54975	543	173	130268	1498	84
**P**	57368	67191	111456	130475	178149	1198	63716	60202	27116	10372	89396	67571	22421	44162	80307	50156	164	26856	2530	162845	15058	36167	12545	2338
**Q**	34335	18486	3070	46732	38361	1037	616	3510	7123	1309	26728	2719	3768	48003	15798	428	951	61	31368	88770	104	106351	508	46
**R**	81840	61491	125283	84055	80404	122492	18693	97899	90432	78688	13754	46545	74256	18039	23368	211054	53548	91410	227242	68354	189288	867	76906	99006
**E**	16773	29939	43	18643	29260	16036	9697	62527	7690	51964	6377	3153	5490	48571	39756	4947	234	13935	18895	233	17536	570	5889	49
**K**	49580	417	2161	36	43836	23804	15997	22	13583	22293	2077	36281	28901	23370	444	124	12246	79	376	240	2091	5797	832	88279
**D**	5348	17956	39488	76398	39021	46	21879	119	29307	948	3317	1957	11711	11179	24477	22399	103284	13602	37417	62531	253	70773	36881	64914
**Stop**	55338	100965	41636	9803	39070	259	16672	2763	1175	148021	281	27004	1908	10413	55873	28058	60738	79832	26096	1198	88853	36	320417	43

B. TM2 Variants in the Presence of 5 µM 9-*cis*-Retinal

	Y74	I75	L76	L77	N78	L79	A80	V81	A82	D83	L84	F85	M86	V87	L88	G89	G90	F91	T92	S93	T94	L95	Y96	T97
**I**	40187	23763	296	9848	13755	7885	40342	15632	38973	4996	36834	20792	11242	24601	7592	38694	14974	131510	81809	1042	8477	207622	24438	25060
**L**	51827	130661	72534	222610	176928	185111	81708	4757	57926	35423	26364	40003	65120	12447	41969	184146	77352	175238	58475	31550	138908	4307	29992	54
**F**	27024	4944	23521	25347	58814	49505	6702	14	25934	16298	6123	2764	18420	292	11827	73524	35410	56677	48258	81029	712	43024	26369	7
**V**	1378	10054	2561	21679	26	8416	7237	56554	26044	35323	67789	41431	20106	24278	33432	616	124065	63156	4234	8555	19551	557	10595	54
**C**	23938	150	24317	74	57475	19875	50070	6677	57480	11363	11854	237	17828	14299	23	493	1148	975	65	15891	2911	22221	590	5392
**M**	9	48901	15509	15	180	1654	24	2069	12150	66	16463	387	0	21681	33168	20	6697	355	249	15	430	61217	172	1
**A**	54253	14341	2000	121902	316	32376	20330	31681	12790	28574	39631	29369	20609	39834	6372	108621	20487	292	31050	1943	80667	129727	28538	125276
**W**	10416	17	58579	0	11	66	4	10055	7833	49649	182	18207	6146	16	8388	1119	51920	62545	7167	45	65825	6552	533	50
**T**	55582	59968	138490	36858	1708	74582	46851	6303	20477	12932	16597	18703	15446	23812	21499	10679	36387	116193	15621	1313	2555	230	9062	2023
**Y**	4628	96718	166	28	603	9846	7503	14	12688	16998	2605	3917	3438	401	61650	138	85519	49502	188	69	10294	44	761	35
**G**	95678	36540	49011	35	13	12	33163	36517	42801	20893	38828	14644	51271	12115	27381	6452	54255	39423	38851	177173	14	50786	33610	17210
**S**	3847	12343	87521	133	132246	95560	53329	32606	43721	37754	53450	17988	62662	53791	63607	61824	99034	37979	129707	43989	108629	6805	25607	1995
**N**	78242	41887	71	17	444	19365	21499	2163	160	13892	13159	44488	20454	51154	194	13871	86	1091	29738	4981	24202	84	19786	1499
**H**	1293	107639	52897	18072	447	71	18301	152	37223	10760	33180	21918	7952	30576	11139	58461	27	9957	47092	473	132	105687	1154	71
**P**	52124	54864	98221	114286	146134	990	56248	47119	23143	8895	81211	61436	20133	35392	63730	45484	153	22936	1975	139815	12501	28110	10066	1618
**Q**	29789	17774	2706	41270	35257	798	643	2985	6019	1476	23385	2490	3105	44840	12789	340	859	50	26806	78165	66	90756	536	30
**R**	72052	50820	101680	72755	55315	109557	17416	64777	78016	64146	9997	32543	62915	13194	15995	150213	42520	66192	164091	53946	126487	765	65246	65116
**E**	15624	27394	22	17316	27415	13186	8274	48707	6962	45934	5453	2739	4369	41283	33660	3701	208	12690	15694	239	15159	453	5076	50
**K**	43471	299	2009	43	28974	20314	15968	23	11968	20930	1645	30362	24790	16125	281	97	9020	63	289	171	1670	3494	571	61213
**D**	4605	16764	35931	66333	34135	34	20123	96	26006	650	2331	2396	11159	8666	21498	18235	87122	11450	33246	55442	229	47063	29460	57793
**Stop**	35582	67911	27756	6793	26991	232	11707	2310	716	99436	221	18221	1530	7795	36777	19780	42769	54954	18202	1057	59537	24	219317	44

*Gray cells indicate variants that were removed due to poor sampling according to the criterion described in the Methods section.

Table S4. Average number of TM7 variant reads across two biological replicates.

A. TM7 Variants in the Absence of Retinal

	F287	M288	T289	I290	P291	A292	F293	F294	A295	K296	S297	A298	A299	I300	Y301	N302	P303	V304	I305	Y306	I307	M308	M309
**I**	20397	11643	22560	25131	23181	33	26101	95908	47173	23619	966	17574	9618	14107	11527	12006	72156	17910	11412	15334	10646	50369	21407
**L**	60906	47437	88713	23090	28053	49431	26336	22338	78086	36604	106786	49939	38095	36730	29266	44776	10933	43940	34539	65842	34544	7749	25475
**F**	13497	10555	44932	5626	11609	4412	2778	1199	58	8407	18441	9596	18699	8211	16353	7675	37888	17780	36484	14748	7126	2759	8820
**V**	21520	36251	3663	29917	24405	54167	25175	42862	93517	67998	28866	45557	44505	33545	38911	45611	40	37006	34228	20997	22938	84804	41015
**C**	310	24774	562	36	12178	10	27969	29928	183	12586	185	21619	4945	12732	12240	15203	6444	14812	51027	17123	18098	163	12770
**M**	121	0	2047	6967	7351	3	6878	10298	2	2161	352	3558	1292	12810	6896	635	59	8408	302	552	1210	0	0
**A**	10413	25705	21542	21096	33196	9213	21775	18628	71973	13937	10735	9837	19504	44835	16791	47394	434	32287	51146	22499	35694	1732	29073
**W**	198	1577	14076	8838	8998	62257	1720	31412	15	8017	179	9250	7182	9806	16817	9103	23430	7020	14364	9351	314	17	6245
**T**	62442	25950	3220	33081	24011	99219	38144	45976	7033	46254	51452	35548	31085	28520	15000	14736	920	29200	24819	43360	25788	707	11074
**Y**	4576	14768	8799	17318	18250	36	14258	33332	16	6269	19418	26857	15007	12559	4869	5586	30112	13865	23442	10213	6632	3202	9189
**G**	54051	41581	32049	66578	31166	33874	32665	49171	23338	26651	31637	42208	37856	33672	52564	63411	78138	41758	31948	33928	24211	80291	29769
**S**	50116	35556	10277	33086	47012	5513	45589	59725	71137	21367	43388	53783	41871	41699	28606	40374	27673	49659	68464	52114	40853	72429	30086
**N**	1041	6535	23605	30103	18968	8771	34342	26624	62	4433	472	9967	7123	5260	6195	4924	31628	15523	30614	15255	4679	452	11743
**H**	5079	15799	6057	5579	8223	105	10603	33371	818	11298	44	21451	7024	18219	7402	17508	10935	17103	23454	23254	24361	2104	17317
**P**	49981	39664	39935	48452	21989	165	45465	42218	2767	53958	31059	53470	52039	51522	21103	35614	2256	29231	43923	54809	38106	37122	30355
**Q**	24445	18949	3007	12181	10466	3885	11394	33874	3807	15729	66	6747	2756	6793	4795	17643	190	28025	16381	16782	12583	183	19260
**R**	59731	61864	97412	54354	44717	6766	40845	122605	16600	58964	18947	51683	44902	34410	31120	53922	73965	70595	86924	19168	55294	55814	45872
**E**	6553	8325	26159	29850	12406	100454	17841	22252	251	15081	83064	15195	5889	10298	13854	2877	9464	19989	20374	17299	13542	33	7748
**K**	29990	18579	19013	29836	28285	25	12135	49631	4	10098	9243	14457	9762	22011	4299	16174	140	14393	37639	8421	20275	9186	9791
**D**	49549	16408	10066	9885	11528	321	20676	27838	447	14072	1552	6526	6957	8572	10262	23886	36	18260	8106	17612	7067	2200	20454
**Stop**	78811	34315	52585	540	28575	3499	37868	54119	21470	24683	3406	73957	39052	25003	21023	27833	29854	40283	36299	34795	24762	76102	5079

B. TM7 Variants in the Presence of 5 µM 9-*cis*-Retinal

	F287	M288	T289	I290	P291	A292	F293	F294	A295	K296	S297	A298	A299	I300	Y301	N302	P303	V304	I305	Y306	I307	M308	M309
**I**	19684	12304	21578	26267	23682	54	24510	93938	48155	23116	1611	18527	8190	14301	11077	11972	70113	20248	11814	15513	12138	48426	22458
**L**	56721	47837	84792	22638	28278	51365	25621	22431	75379	29993	105263	50607	38223	35732	28962	45529	11038	43074	34289	62384	37160	7522	27556
**F**	12768	11803	41552	5725	11440	4518	2817	1282	65	7774	17927	11569	16551	8113	16125	8372	34910	18327	34420	14692	7734	3213	8524
**V**	20770	36248	3785	30751	24012	54502	24473	38129	88963	63123	31860	44634	43260	33161	39300	46899	47	37097	32247	21534	22740	83284	41054
**C**	374	24161	548	46	10893	12	28167	28394	171	11713	168	22524	4364	12923	10267	14415	6688	15567	49842	15984	16324	277	12892
**M**	114	0	1867	7379	7142	1	5722	9660	1	1901	536	4085	1425	13372	5770	745	60	8366	296	462	836	0	0
**A**	10820	24833	22077	20225	30352	9894	21793	19758	68443	15381	11234	9917	21453	41821	16786	45039	568	32852	47773	22106	36115	2089	28602
**W**	118	1432	15027	9067	8179	61862	1849	31301	14	7681	93	9591	7519	8993	16179	7854	21387	7362	15164	9471	291	10	6322
**T**	61061	25477	3962	34287	23416	100443	38010	41657	7179	45396	52206	34034	32231	30023	13809	16276	1136	27799	24599	40594	25960	786	10658
**Y**	3826	12277	8498	19276	17326	47	16710	35132	25	6229	19664	26731	15292	12398	4721	5823	26662	12100	23618	11372	6484	3592	8177
**G**	54201	38781	30471	64466	27553	33162	31472	49680	22312	25875	31790	40801	39360	32794	49080	65525	77065	44636	30246	32038	23964	79985	29045
**S**	49398	35707	12174	34792	46983	6130	45504	58416	76854	20142	43096	54280	42787	37924	28549	37272	28879	48057	67426	48902	41049	70523	31467
**N**	1025	6444	23002	28228	20268	7820	33472	26651	116	4393	568	9483	6092	4675	6881	4238	31207	15508	29497	15105	5414	1083	10886
**H**	5609	13849	6489	4438	7926	201	10186	32572	848	10301	64	21229	6578	16450	8971	19300	11716	14683	21306	22702	21419	2128	20262
**P**	50962	38745	33905	48018	23321	417	45235	43539	3157	52847	31049	56068	50243	44538	20666	34112	2616	29186	45216	52718	39326	31861	24018
**Q**	24397	18810	3011	12322	8982	3722	10448	35113	3964	14063	64	6686	2871	6827	5795	17780	237	21949	14670	17476	12330	214	19293
**R**	55665	57947	95535	51426	43896	6347	34870	112428	14812	56584	17406	52075	40164	31886	30463	49981	68371	68048	75096	18195	55815	52310	44375
**E**	7358	8077	25146	28460	11385	97401	19036	22317	320	13991	77021	17331	5875	9403	14528	3491	8101	16703	17764	17595	11408	39	8633
**K**	31345	16599	17314	27602	26050	50	12975	46550	3	11627	7739	14657	9067	22481	3855	15195	163	12747	33289	9285	17825	10068	8429
**D**	46028	14307	10391	9891	11559	404	20493	26403	558	13343	1772	6556	6934	7957	9812	22632	33	17211	7345	18101	5827	2041	20781
**Stop**	74206	36355	51107	793	26929	3093	36087	52862	17875	22687	2543	69282	35850	22884	19722	28526	27504	38769	34770	33002	22616	71451	4520

*Gray cells indicate variants that were removed due to poor sampling according to the criterion described in the Methods section.
